# Understanding and harnessing intergroup contact in educational contexts

**DOI:** 10.1111/bjso.12876

**Published:** 2025-03-07

**Authors:** Shelley McKeown, Loris Vezzali, Sofia Stathi

**Affiliations:** ^1^ Department of Experimental Psychology University of Oxford Oxford UK; ^2^ Faculty of Medicine University of Modena and Reggio Emilia Reggio Emilia Italy; ^3^ Institute for Lifecourse Development, Centre for Inequalities University of Greenwich London UK

**Keywords:** education, intergroup contact, intervention, policy, prejudice reduction, schools, teachers

## Abstract

Prejudice is a pervasive problem that affects each and every one of us. Understanding how to reduce prejudice and promote better outcomes for both individuals and societies at large is an ambitious but essential task. For decades, social psychologists have theorized about and evaluated approaches to achieve just that, and there is one that stands out from the rest: facilitating intergroup contact, that is, (positive) interactions between members of different groups. Questions remain, however, about how and where good quality (meaningful and cooperative) interactions can be promoted in the face of societal division, and whether such interactions can foster social equality. In this paper, we argue for the importance of educational contexts as sites where future generations encounter the opportunity to interact with, or at the very least learn about, people who are different from them. We first outline social psychological research on the nature and effects of having frequent and good quality contact with people who are different from us, demonstrating evidence from education settings globally. We then provide a series of recommendations for schools and teachers on how to reduce prejudice in the classroom in both the presence and absence of difference.

Prejudice is deeply entrenched in societies, affecting the lives of many children and young people growing up today. Understanding how to best reduce prejudice and build socially just societies for future generations is an ambitious but essential task. A key question, however, is how we might achieve this. Over several decades, social psychologists have developed robust scientific tests of approaches to prejudice reduction, and there is one in particular that stands out for its potential: facilitating frequent positive and meaningful interactions between groups. Here, we focus on the role that educational contexts can play as places where young people have the opportunity to interact with people who are different from them, across dimensions such as social class, ability and ethnicity, and where interventions can be implemented. We outline research on the nature and effects of intergroup contact in educational settings. In doing so, we caution against contact as the sole solution to tackling prejudice and building more equitable societies. We then provide a series of theory‐informed recommendations for schools and teachers on how to reduce prejudice in their classrooms.

## WHAT IS INTERGROUP CONTACT AND WHY DOES IT MATTER?

Put simply, intergroup contact is the premise that positive and meaningful interactions with individuals from a different group, whether that be race, religion, sexuality, ability, etc., can lead to feeling less prejudice towards that group. In his 1954 book, *The Nature of Prejudice*, Gordon Allport specified that four conditions need to be in place for contact to reduce prejudice — and such conditions are arguably particularly possible within educational contexts. First, there should be equal status within the contact situation between the groups that are interacting (e.g., in the school classroom). Second, there should be cooperation between the groups; and third, the groups should be working towards common goals (e.g., students working together on a school project). And finally, there should be social or sanctioning support for the contact (e.g., teachers/schools encouraging interaction between group members).

Since its inception, research on intergroup contact has grown exponentially, with a wide range of studies conducted amongst samples across ages and around the globe on how both direct (face‐to face) and indirect forms of contact (e.g., extended and vicarious contact) are associated with outcomes related to prejudice reduction and intergroup relations more broadly (Dovidio et al., 2011; White et al., 2021; for meta‐analysis see Pettigrew & Tropp, 2006). Contact is said to work mainly through affective factors, such as encouraging empathy and reducing feelings of anxiety about interacting with people from other groups (Pettigrew & Tropp, 2008); but it also works through cognitive mechanisms, like increasing knowledge about people from other groups (Pettigrew & Tropp, 2008) or changing group representations from “us” and “them” to a common “We” (Gaertner & Dovidio, 2000). Evidence shows that contact can reduce prejudice even for highly prejudiced individuals (Turner et al., 2020). It is perhaps unsurprising, therefore, that the principles of intergroup contact are often applied within educational contexts, with the goal of building stronger relations between socially divided, conflicting and stigmatised groups.

## INTERGROUP CONTACT IN EDUCATION

Research generally shows that direct forms of intergroup contact amongst children and young people attending educational contexts are associated with more positive social attitudes (Brown et al., 2007; De Tezanos‐Pinto et al., 2010). Survey studies, for example, have demonstrated that more frequent intergroup contact with minoritized groups is associated with lower levels of prejudice and more supportive attitudes towards minority rights amongst youth in Catalonia (Wilson‐Daily et al., 2018), that holding more cross‐group friendships is associated with less extreme political attitudes in Northern Ireland (Stringer et al., 2009), and that higher quality intergroup contact is associated with more support for peacebuilding and, in turn, more positive societal engagement in Northern Ireland (McKeown & Taylor, 2018). There is also evidence that intergroup contact is effective in attenuating teachers' biases too (Doyle et al., 2024; Yang et al., 2023). These studies, however, due to their correlational design, are unable to conclude that contact experiences *cause* changes in attitudes. That said, there is evidence of the beneficial effects of contact‐based interventions in educational contexts using field and natural experimental designs (Aboud et al., 2012; Tropp et al., 2022; Ülger et al., 2018).

In Northern Ireland, for example, Reimer et al. (2022) found that youth who attended shared classes between Protestants and Catholics (based on a contact‐focussed education programme) reported having more contact out of school, more positive attitudes, outgroup trust, and empathy compared to those who did not share classes as part of the programme. In the US, Shook and Fazio (2008) found that university students randomly assigned to different‐race roommates showed lower levels of prejudice and intergroup anxiety towards outgroup members later in the semester compared to the start of the semester, whilst no change was observed for same‐race roommates. There is also experimental work examining how to get the most out of educational contact interventions. Vezzali et al. (2023), for example, examined the role of categorisation in intergroup contact through two direct contact field experiments in schools in Italy and found that contact effects can be maximized when emphasizing group categorisation (i.e., having distinct identities) first and then moving to decategorisation (i.e., minimize group/identity distinctions).

Contact in educational settings, however, is not always direct. There is evidence that indirect forms of intergroup contact can have positive effects (see Di Bernardo et al., 2017 for a review). In a series of experimental studies, for example, it was found that storybook reading (Cameron & Rutland, 2006) and video watching (Cocco et al., 2021), where stories of intergroup friendships were created ad‐hoc or selected from published fiction, were associated with more positive attitudes towards outgroups compared to control conditions. The simulation of intergroup interactions ‘in the mind's eye’ (imagined contact) has also been used successfully in school interventions (Stathi et al., 2014).

## BEING CAUTIOUS OF CONTACT'S POTENTIAL IN EDUCATIONAL CONTEXTS

Despite evidence in support of intergroup contact's positive effects, it is important to remain cautious about its potential to build more equitable societies. When it comes to considering application in educational contexts specifically, we argue that there are three main points of caution for researchers and educators to keep in mind.

First, it should be recognized that the opportunity for contact (e.g., being in the same classroom as people from another ethnic group) does not necessarily mean that (meaningful) contact will actually occur (Dixon & McKeown, 2021). Indeed, behavioural mapping studies conducted in schools in England (Al Ramiah et al., 2015; McKeown et al., 2017) and in Northern Ireland (McKeown et al., 2015) demonstrated that children and young people often remain segregated across group lines, suggesting that physical co‐presence does not necessarily lead to meaningful interaction in school settings.

Second, it should be noted that contact experiences are often quite banal, that they may even be negative, and could potentially backfire, especially in deeply divided societies, where relations are unequal and fraught (Dixon & McKeown, 2021). Research shows, for example, that negative contact is more strongly associated with prejudice than positive contact is with prejudice reduction (Barlow et al., 2012; Paolini et al., 2024). It has also been found that contact may have differential effects for advantaged and disadvantaged groups (Tropp & Pettigrew, 2005), and that disadvantaged groups may be less likely to engage in actions to improve their position in society following (positive) contact with advantaged group members (Saguy et al., 2009). Bringing young people from conflicting groups together in highly charged situations, therefore, requires careful consideration and in fact, it might not always be appropriate.

Third, it is essential to consider that there have been mixed results when it comes to the effects of contact‐based field studies on prejudice and related outcomes (Mousa, 2020; Paluck & Green, 2009). A significant proportion of evidence for contact effects is based on cross‐sectional findings, and contemporary longitudinal evidence questions whether contact does indeed lead to individual changes or rather that it simply is that people who report more contact also tend to report lower levels of prejudice (Hodson & Meleady, 2024; Sengupta et al., 2023). This is directly relevant to educational contexts given that Friehs et al. (2024), for example, were unable to find evidence that cross‐ethnic friendships in school were associated with individual changes in outgroup attitudes over time.

## RECOMMENDATIONS FOR EDUCATION‐FOCUSSED RESEARCH, POLICY, AND PRACTICE

Whilst caution is important, there is in our view sufficient evidence from educational contexts that intergroup contact can be associated with more positive outcomes for youth (e.g., De Tezanos‐Pinto et al., 2010; Reimer et al., 2022) with implications for society at large. We therefore offer a series of recommendations for researchers and educationalists to get the most out of the potential of intergroup contact in practice relevant for a range of group divides.

Our first recommendation is that support is needed to equip teachers and schools with the skills to know when and how to encourage positive and buffer potential negative interactions. This could be achieved by promoting research‐informed practice through: knowledge exchange visits, the development of resources for teachers to use in their classrooms, and the inclusion of intergroup relations modules within teacher training programmes. This way, academics and practitioners could benefit from a deeper exchange of ideas and expertise (Schalet et al., 2020). We have previously, for example, worked with religious education teachers to design a teaching guide based on the principles of intergroup contact theory (Christopher et al., 2018) but this approach could be equally applied to promoting inclusion based on other divides, such as ability.

Our second recommendation is that when designing contact‐based interventions, researchers and educators should consider not only how to encourage contact but also how to do so in a way that promotes the contact conditions outlined by Allport (1954) alongside facilitating meaningful dialogue. This could include, for example, encouraging students to work on collaborative projects while promoting equal status and supporting cross‐group interactions. Such collective approaches convey the message that the teacher and the school more broadly are committed to diversity, equality and inclusion (DEI), for all groups, ensuring institutional support (Pettigrew & Tropp, 2006). Incorporating debates on cross‐group issues, communication from experts, as well as relevant documentaries and books into the curriculum may also further facilitate meaningful interactions. Such initiatives may help increase educators' and young people's DEI literacy and confidence, allowing for more meaningful dialogue on issues that can be otherwise overlooked due to being difficult or uncomfortable. Research shows, for example, that discussing ethnic differences was associated with reductions in segregation seating behaviour amongst children in England and young people in Northern Ireland (McKeown et al., 2012, 2017).

Our third recommendation is that contact interventions in educational contexts should both go beyond single or relatively short‐lived programmes (e.g., 3–4 sessions), a common limitation of field interventions (Paluck et al., 2021), and be context‐sensitive. In contexts of high conflict, for example, indirect contact in educational contexts can offer an opportunity to reduce anxiety before direct contact takes place in the wider community. Contact interventions should, therefore, be viewed as part of a collective toolkit. Running complementary direct and indirect contact interventions throughout the school year(s) should strengthen their individual efficacy (see Vezzali et al., 2019 for an example of an intervention combining direct and vicarious contact approaches). Interventions can also be applied to address inequalities faced by multiple groups (e.g., social class, ability, ethnicity and more).

Our fourth recommendation is that researchers and educators work in collaboration to develop an agenda for implementing and evaluating the role of intergroup contact in education. The experience of school teachers and managers, who know activities typically conducted in schools and the school resources, coupled with the expertise of researchers on state‐of‐the‐art psychological theories and processes, can better inform robust interventions. Indeed, evidence shows that prejudice‐reduction intervention effectiveness is stronger when activities are research‐informed (Ülger et al., 2018), and when researchers and teachers work together (Cameron & Rutland, 2016). We argue, therefore, that this researcher‐practitioner connection is essential for improving intergroup relations in divided societies (Stephan, 2006) and should be central to designing contact interventions in educational contexts, regardless of the form of prejudice that educators are wishing to tackle.

## CONCLUSION

It is crucial now to do what we can as researchers, educators, and practitioners to reduce prejudice and build stronger and more equal societies. Promoting intergroup contact is arguably one of the most successful ways to work towards this goal. Contact, however, should not be seen as a panacea to tackling the large‐scale inequalities that we see across social class, ability and ethnic divides. We argue that promoting different forms of contact in parallel with other approaches, and doing so in collaboration with researchers and practitioners, is essential for the development and implementation of successful and sustainable interventions. It is now time to create a research‐practice agenda that puts theory to the test where it is needed the most — in the field.

## AUTHOR CONTRIBUTIONS

**Shelley McKeown:** Conceptualization; writing – original draft; writing – review and editing. **Loris Vezzali:** Writing – original draft; writing – review and editing. **Sofia Stathi:** Conceptualization; writing – original draft; writing – review and editing.

## Data Availability

There are no data associated with this manuscript.
